# Tear Ferning Test in Sheep: Scale‐Based Analysis, STEPanizer Method, and Correlation With Conjunctival Histological Findings

**DOI:** 10.1111/vop.70301

**Published:** 2026-11-09

**Authors:** Aline Stephanie Silva Carvalho, Brendha Mendes Ferreira, Juliano Pereira Terra, João Vitor Viana Borges, Fabiano José Ferreira Sant'Ana, Paulo Henrique Sampaio Silva, Paula Diniz Galera

**Affiliations:** ^1^ Faculty of Agronomy and Veterinary Medicine University of Brasília Brasília Brazil; ^2^ Self‐Employed Veterinarian Brasília Brazil

**Keywords:** conjunctival inflammation, goblet cells, sheep, STEPanizer, tear ferning test, tear film

## Abstract

**Objective:**

To evaluate the precorneal tear film (PTF) of clinically healthy sheep using the tear ferning test (TFT) and investigate its association with conjunctival histological parameters.

**Animals Studied:**

One hundred and thirty‐four eyes from 67 adult sheep.

**Procedures:**

Tear samples were collected and graded by three evaluators using the Rolando and Masmali grading scales. Digital stereological analysis of tear ferning patterns was performed using STEPanizer to quantify crystalline points. Conjunctival histology included assessment of inflammatory cell infiltration and goblet cell (GC) density. Data were analyzed using descriptive statistics, Spearman's correlation, Kendall's coefficient of concordance, and simple linear regression.

**Results:**

Rolando and Masmali grading scales showed strong positive correlations between right and left eyes (*r* = 0.922 and 0.886, respectively; *p* < 0.0001), demonstrating high consistency between eyes. STEPanizer crystal point counts were strongly and negatively correlated with TFT grades on both scales (*r* = −0.826 to −0.890; *p* < 0.0001). Linear regression showed that stereological measurements explained 66%–83% of the variation in TFT grades (*R* ^2^ = 0.66–0.83). Interobserver agreement was acceptable (Kendall's W = 0.69; *p* < 0.05). No significant correlations were found between TFT grades and histological parameters. Mild lymphocytic infiltration predominated (83.96%), while only weak negative associations were observed between TFT grades, GC density, and inflammatory infiltrate.

**Conclusions:**

In clinically healthy sheep, TFT patterns appear to be influenced primarily by tear film physicochemical characteristics rather than routine conjunctival histological findings. Combining TFT with digital stereological analysis provides an objective and feasible approach for PTF characterization in veterinary ophthalmology.

## 1 Introduction

The ocular surface (OS) is composed of several structures that, in perfect balance, maintain the health of the cornea, eyelids, conjunctiva, third eyelid, and lacrimal system. The precorneal tear film (PTF), an essential component of the OS, consists of a superficial aqueous‐lipid layer and an inner mucin layer [1]. The tear film is produced by the main and accessory lacrimal glands, conjunctival goblet cells (GC), and the meibomian glands located in the upper and lower tarsal plates [2].

The bilaminar structure of the PTF is essential for ocular surface homeostasis [3]. The lipid component of the aqueous‐lipid layer is secreted mainly by the meibomian glands, reduces evaporation, stabilizes the air‐tear interface, and facilitates smooth eyelid motion [4]. The aqueous component, produced by the main and accessory lacrimal glands, provides nutrients, antimicrobial factors, and bulk fluid necessary for debris removal. The inner mucin layer, derived primarily from conjunctival goblet cells, promotes wettability of the corneal epithelium, anchors the tear film to the ocular surface, and contributes to its optical regularity [5].

A high‐quality PTF supports the health of the OS structures and, conversely, OS integrity influences PTF stability. A stable tear film ensures an effective refractive medium and ocular comfort [2]. Alterations in any of these layers, age‐related changes [6] and environmental stressors such as high temperature and low humidity [7] have been associated with increased tear osmolarity and ocular surface inflammation in both humans and experimental animal models [8, 9].

The Tear Ferning Test (TFT) is a qualitative tear evaluation method that has recently gained attention in veterinary medicine but is not yet widely applied in routine clinical practice. TFT has been described in several animal species, often in correlation with other tear film tests, including dogs [10], cats [11], horses [12], rabbits [13], capuchin monkeys [14], birds and reptiles [15]. In sheep, the effect of electrolyte addition on TFT has been reported [16].

Two main grading systems have been described for the classification of the TFT. Rolando's original grading system classifies ferning patterns into four types (I–IV) according to branch density, homogeneity and the presence of amorphous areas, with Types I–II representing normal tears and Types III–IV indicating tear film disturbance [17]. In Types I–II patterns, densely packed, long and regularly branching ferns cover most of the dried droplet, whereas Types III–IV show shorter, fragmented branches with large amorphous or crystal‐free areas, reflecting impaired crystallization. In contrast, Masmali et al. proposed a 0–4 grading scale with more detailed morphological criteria, which has shown higher repeatability and inter‐observer agreement in human studies and has been increasingly adopted in veterinary species. In this system, grades 0–1 display uniform, well‐branched ferns with minimal spaces between crystals, while grades 3–4 are characterized by sparse, non‐uniform aggregates, disrupted branching and extensive amorphous zones [18]. Using both grading systems in parallel may therefore improve comparability with previous human and veterinary studies and allow a more nuanced characterization of ferning patterns in sheep.

The TFT is a low‐cost, rapid, and highly accurate method for diagnosing tear film disorders such as DED and has also been proposed as a predictive factor for tolerance and successful contact lens wear [19, 20]. Tear sample collection methods vary from pipette aspiration, Schirmer Tear Test (STT) strips, and spatulas to capillary tubes, the latter being preferred for better repeatability and less variation in sample volume [18, 21, 22]. The ferning pattern is influenced by tear film composition and environmental factors such as temperature and humidity [18, 21, 22, 23, 24]. Classification is performed using grading scales that assess the density and branching of the ferns, thus reducing subjectivity [17, 25].

The STEPanizer software applies stereological principles to microscopic and macroscopic images, enabling three‐dimensional quantitative analysis from two‐dimensional images, reducing the subjectivity of visual evaluation and increasing reproducibility across experimental groups [26, 27, 28, 29].

The objective of the study was to describe TFT in sheep, correlating it with the temperature and humidity conditions of the Central‐West region of Brazil, the animals' age, and employing a technological approach. This approach based on the combined use of the Rolando [17] and Masmali [18] scales in conjunction with digital stereological analysis provided by the STEPanizer software. Additionally, the study aimed to correlate the TFT with the histological characteristics of the palpebral conjunctiva, including inflammatory cell infiltration and GC counts, as well as to explore its influence on PTF dynamics.

## 2 Materials and Methods

### 2.1 Animals

All animals underwent an ophthalmic examination before inclusion in the study. The examination was performed by a trained veterinary ophthalmologist in a shaded area of the pen using a head‐mounted magnifying loupe and a focal light source. The evaluation included assessment of menace response, dazzle reflex, and pupillary light reflexes, as well as inspection of the eyelids, conjunctiva, third eyelid, and cornea. Particular attention was given to the presence of discharge, conjunctival hyperemia, chemosis, corneal opacity, or neovascularization. No Schirmer Tear Test (STT) was performed in order to avoid interference with subsequent tear sampling for TFT.

Sheep with any ophthalmic or systemic disease and environmental temperature and humidity fluctuations that compromised tear samples were excluded. Therefore, a total of 134 eyes from 67 sheep were included in the analysis. The animals were crossbreeds of Morada Nova, Santa Inês, Dorper and White Dorper, from the Água Limpa Farm, University of Brasília, housed in covered pens under identical management and feeding conditions, with regional standard temperature and humidity.

Owner consent was not applicable because all sheep belonged to the institutional experimental flock and were managed under the approved animal use protocol. This study was approved by the Institutional Animal Care and Use Committee of the University of Brasília (Protocol no. 23106.068874/2024‐67).

### 2.2 Tear Ferning Test (TFT)

Tear film collection and ferning pattern analysis were performed as previously described [12] and were performed by a single examiner in the same environment. Sampling took place in the animals' pen, with gentle physical restraint in a chute by a trained technician. Tears were collected from the lower palpebral meniscus of both eyes using a capillary tube and deposited onto identified and pre‐marked glass slides (3 mm diameter circle) to standardize location and volume. Slides were stored in a climate‐controlled room until complete drying, then examined under a light microscope with a coupled camera for image acquisition. Ambient temperature and humidity were recorded using a digital thermo‐hygrometer (Incoterm Thermometer Industry Ltd., Porto Alegre, RS, Brazil).

Images were analyzed using STEPanizer stereology software to quantify crystalline points (36‐point standardized grid). Points coinciding with crystals were scored positive. Ferning patterns were graded independently by three trained veterinary ophthalmologists using Rolando's [17] and Masmali's [18] scales, at 200× magnification. All three examiners had previously undergone standardized training for TFT grading in dogs prior to this study.

### 2.3 Goblet Cell Analysis of the Palpebral Conjunctiva

Biopsies of the nasal inferior conjunctival fornix were performed as previously described [12], by the same examiner, on three separate days within one month, under similar environmental conditions, always in the morning. Prior to sampling, the conjunctiva was treated with 1% povidone–iodine for 15 s [30]. This was followed by instillation of one drop of topical anesthetic (tetracaine hydrochloride 1% with phenylephrine hydrochloride 0.1%; Allergan, SP, Brazil), which was considered sufficient to achieve surface anesthesia for the brief biopsy procedure and to reduce handling stress. No clinically relevant conjunctival reactions, such as hyperemia, chemosis, or discharge, were observed following the application of either agent. Samples (~2 mm) were fixed in 10% neutral‐buffered formalin and processed using routine protocols. Sections were stained with hematoxylin–eosin (HE) and periodic acid‐Schiff (PAS).

Histological evaluation included GC counts in five consecutive epithelial fields at 400× magnification. Inflammatory infiltrates were graded on a three‐point scale by two independent examiners: mild [1], defined as a small number of inflammatory cells scattered throughout the stroma, without aggregate formation or architectural disruption of the epithelium; moderate [2], characterized by a moderate number of cells with small focal aggregates in the superficial stroma and possible mild epithelial thickening; and severe [3], defined as dense, diffuse inflammatory infiltrates or large aggregates, potentially accompanied by lymphoid follicles, epithelial erosion or goblet cell loss [12, 31]. The predominant inflammatory cell type was also identified in each sample.

Analyses were performed independently by two veterinary ophthalmologists, both of whom underwent standardized training in conjunctival histological evaluation at the Veterinary Pathology Diagnostic Laboratory (LDPV/UnB) prior to the study. Mean values between examiners were used for final analysis.

### 2.4 Statistical Analysis

Data was analyzed using GraphPad Prism (v.10, GraphPad Software, MacOS, 2023). Frequency analysis was used to describe TFT grades according to Rolando [17] and Masmali [18] scales. The Shapiro–Wilk test assessed data normality. The Wilcoxon test compared STEPanizer point counts between right and left eye. Spearman's correlation assessed relationships between STEPanizer counts, TFT grades, and between grading scales. Linear regression modeled the association between point counts and TFT grades. Interobserver agreement was assessed using Kendall's coefficient of concordance. Statistical significance was set at *p* < 0.05.

## 3 Results

A total of 134 eyes from 67 sheep were analyzed, comprising 88% females and 12% males, with a mean age of 2 years and 10 months (±1 year and 11 months) (Table 1). The animals were crossbreeds of Morada Nova, White Dorper, Dorper, and Santa Ines, which are among the most common breeds in Brazil [32]. TFT samples were dried under controlled temperature and humidity conditions, with mean values of 21.5°C and 49.5%, respectively.

**TABLE 1:** Distribution of sheep according to sex and age (years).

Sex	Age (years)	*n* animals	% of total
F	2–8	59	88.1
M	1–2	8	11.9
Total		67	100.0

*Note:* Age refers to full years at the time of sampling.

Abbreviations: F, female; M, male.

According to Rolando's scale [17], the most frequently observed types were I and III (Table 2). In the right eye (OD), Type I accounted for 33.33% and Type III for 31.75%, whereas in the left eye (OS), Type III accounted for 35.56% and Type I for 29.63%. Type IV was the least observed in both eyes. Using Masmali's scale [18], Grade 3 predominated in both eyes, with frequencies of 32.56% (OD) and 44.78% (OS). Grade 4 was the least frequent, with 4.25% (OD) and 5.97% (OS). In OD, Grades 2, 1, and 0 occurred in 30.19%, 23.40%, and 9.43% of eyes, respectively; in OS, these grades occurred in 19.40%, 16.42%, and 13.43%, respectively. A strong positive correlation was found between the Rolando [17] and Masmali [18] scales for OD (*r* = 0.922; *p* < 0.0001) and OS (r = 0.886; *p* < 0.0001) (Table 2) [33, 34], supporting the reliability of the classifications and validating the concurrent use of both scales. Finally, the crystalline morphology observed in sheep (Figures 1 and 2) resembled that previously reported in dogs [10], cats [11] and horses [12]. Interobserver agreement was evaluated using Kendall's coefficient of concordance and was considered acceptable (W = 0.69; *p* < 0.05).

**TABLE 2:** Frequency comparison of tear ferning test scores in each eye according to Rolando [17] and Masmali [18] scales and Spearman's correlation (*r*) between scales.

Classification	Rolando scale		Masmali scale		Correlation (*r*)^a^
Right Eye (OD)	I	33.33%	0	9.43%	0.922 (*p* < 0.0001)
	II	20.63%	1	23.40%	
	III	31.75%	2	30.19%	
	IV	10.94%	3	32.56%	
			4	4.25%	
Left Eye (OS)	I	29.63%	0	13.43%	0.886 (*p* < 0.0001)
	II	22.22%	1	16.42%	
	III	35.56%	2	19.40%	
	IV	12.96%	3	44.78%	
			4	5.97%	

^a^ *r* = Spearman's correlation coefficient quantifying the association between Rolando and Masmali tear ferning scale scores for each eye.

**FIGURE 1:**
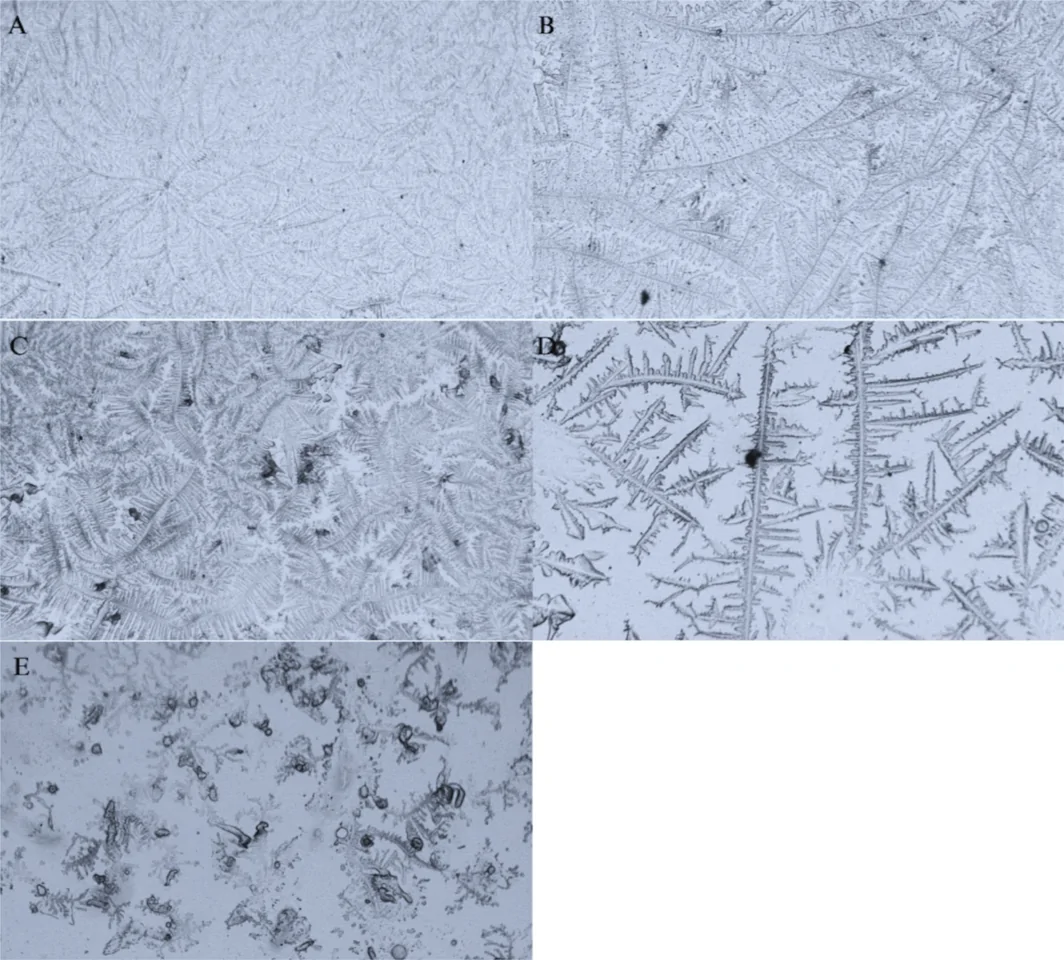
Tear crystal ferning patterns in sheep graded according to the Rolando and Masmali scales, respectively: (A) Type I and Grade 0; (B) Type I and Grade 1; (C) Type II and Grade 2; (D) Type III and Grade 3; (E) Type IV and Grade 4. Magnification 100×.

**FIGURE 2:**
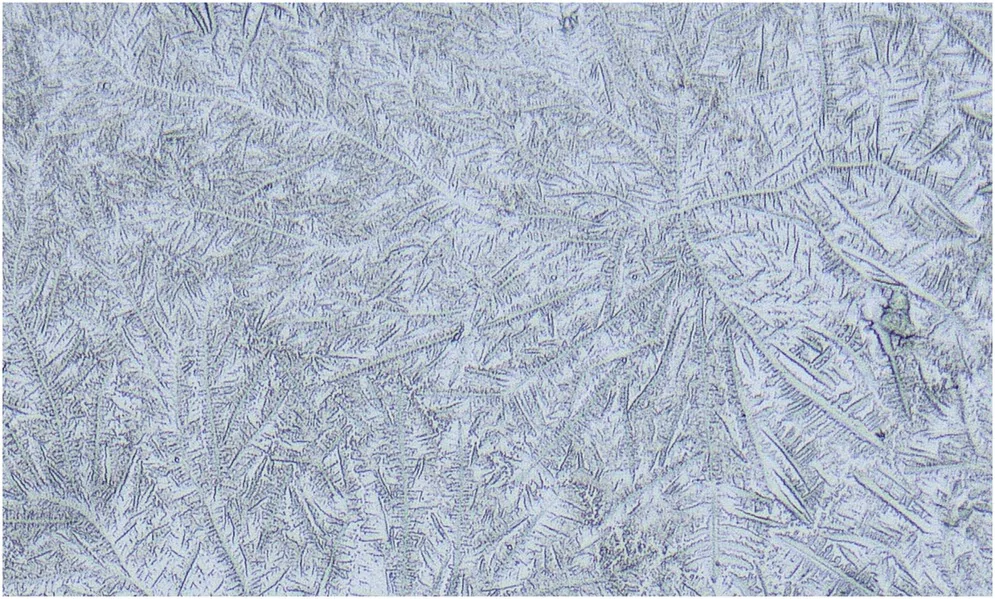
Tear crystal ferning pattern of a sheep graded as Type I and Grade 0 according to the Rolando and Masmali scales, respectively. Magnification 100×.

The STEPanizer analysis revealed a median point count of 22.30 (95% CI: 20.00–24.60) for the right eye (OD) and 20.87 (95% CI: 18.47–23.26) for the left eye (OS) (Table 3). No significant difference was found between eyes for STEPanizer point counts (*p* = 0.2495). Significant negative correlations were observed between STEPanizer point counts and TFT grades on Rolando's scale for OD (*r* = −0.862; *p* < 0.0001) and OS (*r* = −0.826; *p* < 0.0001), and on Masmali's scale for OD (*r* = −0.890; *p* < 0.0001) and OS (*r* = −0.889; *p* < 0.0001). Linear regression models further demonstrated that STEPanizer point counts explained a large proportion of the variance in TFT grades, yielding the following equations: TFT ROL OD = 4.350–0.09334 × POINTS (*R* ^2^ = 0.76; *p* < 0.0001); TFT ROL OS = 4.183–0.08317 × POINTS (*R* ^2^ = 0.66; *p* < 0.0001); TFT MAS OD = 4.522–0.1124 × POINTS (*R* ^2^ = 0.83; *p* < 0.0001); and TFT MAS OS = 4.337–0.1055 × POINTS (*R* ^2^ = 0.77; *p* < 0.0001) (Table 3; Figure 3). No statistically significant associations were found between age and TFT grading scales or STEPanizer analysis (Figure 4).

**TABLE 3:** Correlation between Rolando and Masmali tear ferning scales and median STEPanizer point counts in right (OD) and left (OS) eyes.

Scale	Eye	Correlation (*r*)	Linear regression (TFT = a + b × POINTS)	*R* ^2^	*p*
Rolando	OD	−0.862	TFT ROL OD = 4.350–0.09334 × POINTS	0.7600	*p* < 0.0001
	OS	−0.826	TFT ROL OS = 4.183–0.08317 × POINTS	0.6634	*p* < 0.0001
Masmali	OD	−0.890	TFT MAS OD = 4.522–0.1124 × POINTS	0.8300	*p* < 0.0001
	OS	−0.889	TFT MAS OS = 4.337–0.1055 × POINTS	0.7700	*p* < 0.0001
Correlation between scales	OD	0.922	—	—	*p* < 0.0001
	OS	0.886	—	—	*p* < 0.0001

*Note:* Positive values of *r* indicate direct correlations; negative values indicate inverse correlations.

Abbreviations: POINTS, median number of STEPanizer points; *r*, Spearman's correlation coefficient; TFT, tear ferning test.

**FIGURE 3:**
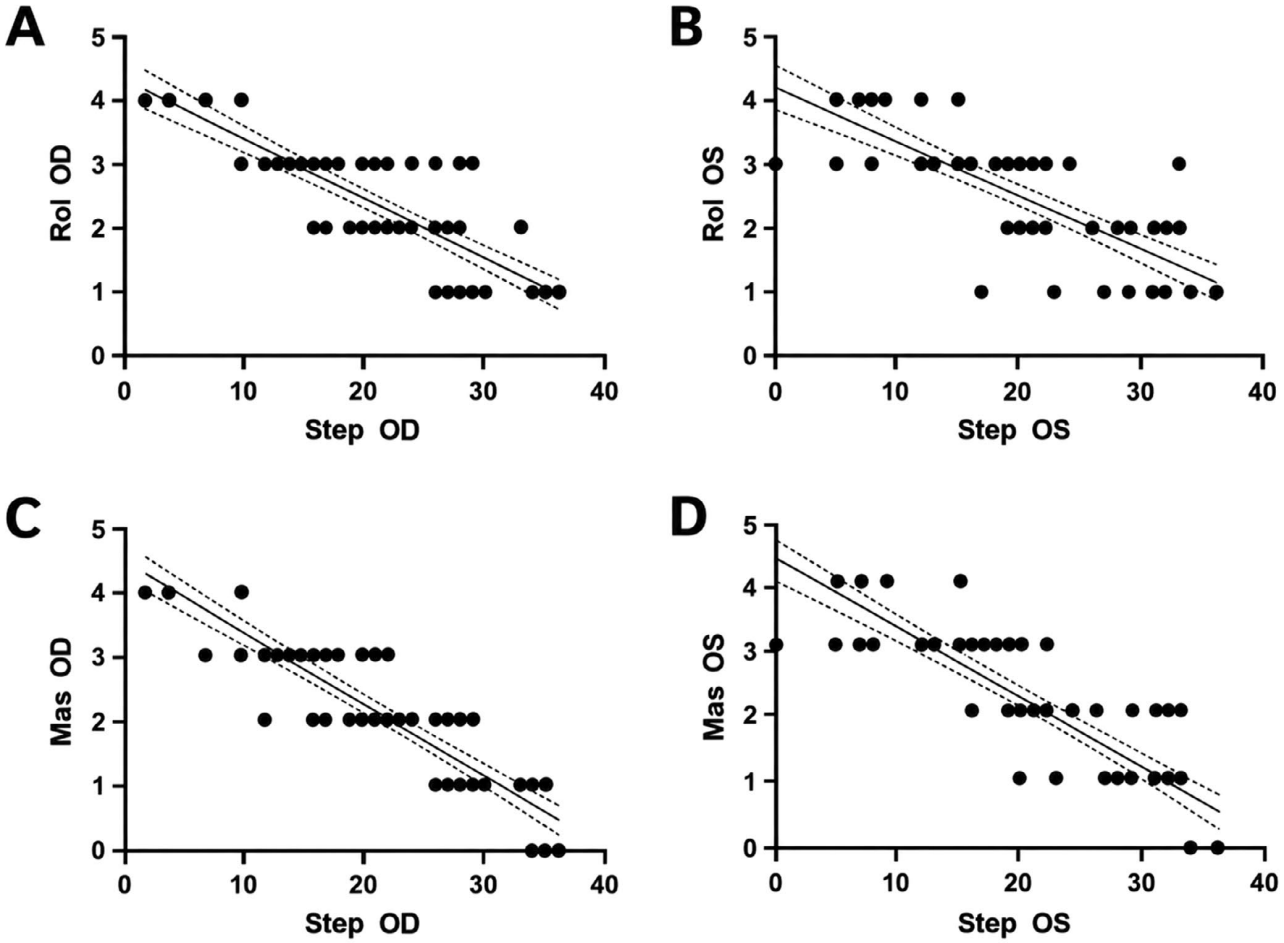
Linear regression between STEPanizer point counts and tear ferning grades according to the Rolando scale in OD (A) and OS (B), and the Masmali scale in OD (C) and OS (D).

**FIGURE 4:**
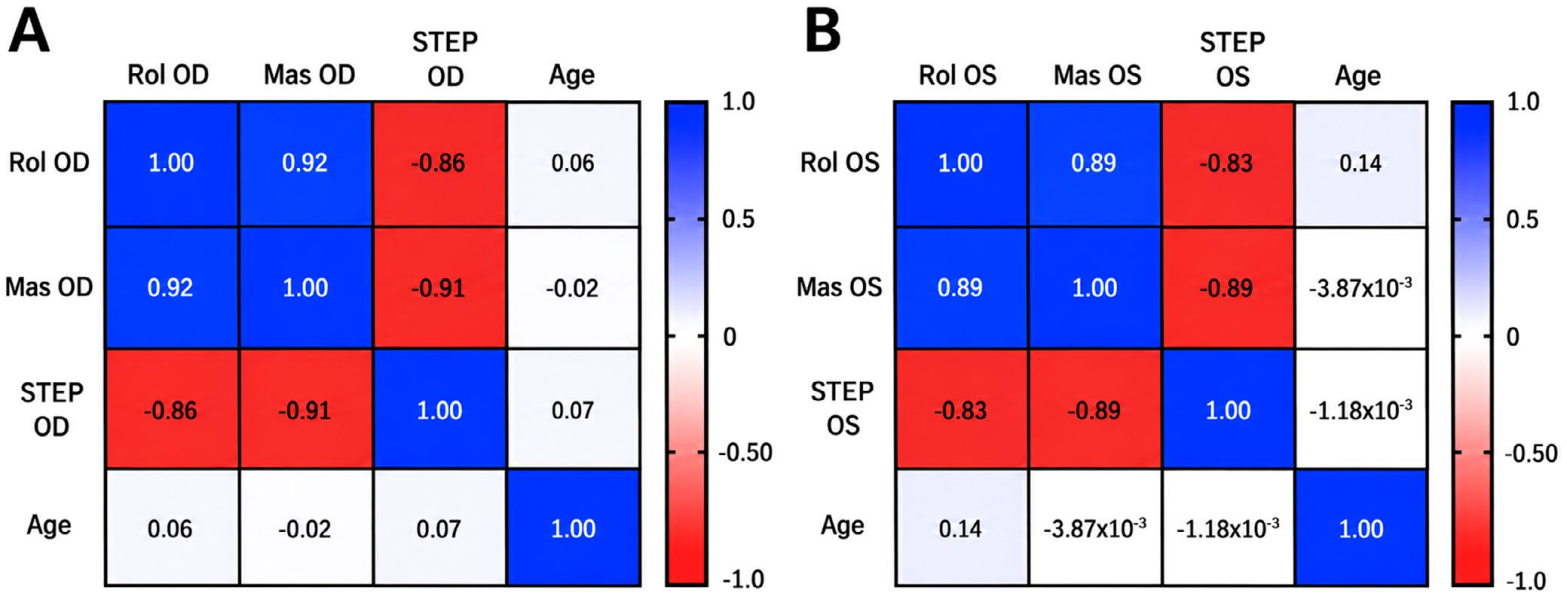
Heatmaps showing the correlations between Rolando and Masmali tear ferning grades, STEPanizer point counts and age in OD (A) and OS (B).

No significant correlation was observed between TFT and histological findings (Table 4). Spearman's correlation showed a weak negative association between Rolando's grades [17] and GC count (*r* = −0.176; *p* = 0.167) and inflammatory infiltrate (*r* = −0.024; *p* = 0.849), and between Masmali's scale [18] and GC count (*r* = −0.059; *p* = 0.644) and inflammatory infiltrate (*r* = −0.058; *p* = 0.652). No significant correlation was observed between GC count and inflammatory infiltrate (*r* = 0.182; *p* = 0.152). A predominance of lymphocytic inflammatory infiltrate was found in 83.96% of samples, with lymphoplasmacytic infiltrate as the second most frequent type (Figure 5).

**TABLE 4:** Spearman correlation between Rolando and Masmali tear ferning grades, goblet cell counts and inflammatory infiltrate of the palpebral conjunctiva in sheep.

Correlation	*r* (Spearman's ρ)	*p*	Significance
Rolando versus Masmali	0.720	< 0.0001	Significant
Rolando versus CGs	−0.176	0.167	NS
Rolando versus inflammatory infiltrate	−0.024	0.849	NS
Masmali versus CGs	−0.059	0.644	NS
Masmali versus inflammatory infiltrate	−0.058	0.652	NS
CGs versus inflammatory infiltrate	0.182	0.152	NS

Abbreviations: CGs, goblet cells; NS, not significant; *r*, Spearman's correlation coefficient.

**FIGURE 5:**
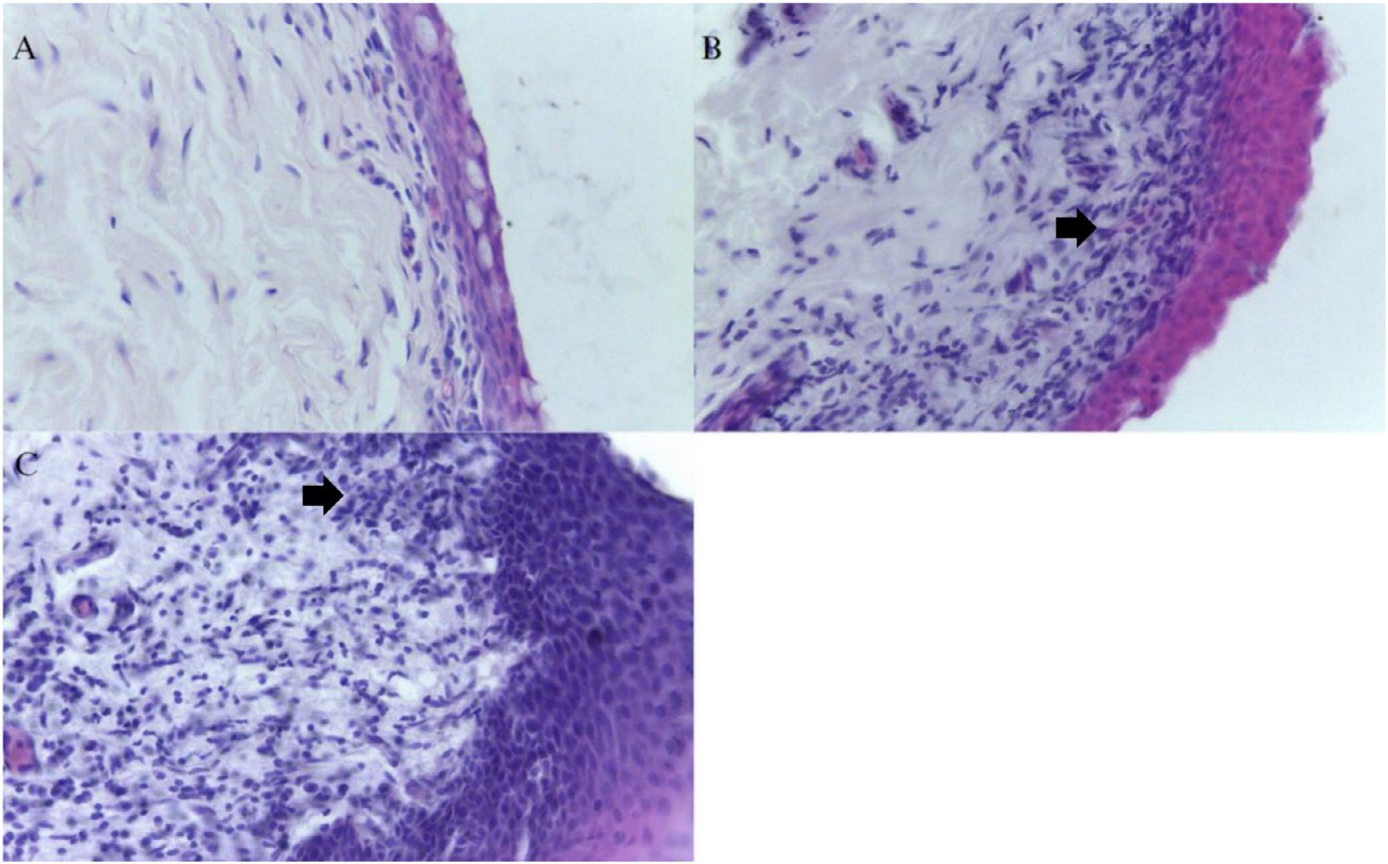
Histological sections of the palpebral conjunctiva classified according to the intensity of inflammatory infiltrate: (A) mild; (B) moderate; (C) marked. Arrows indicate representative areas of inflammatory cell infiltration. H&E staining. Magnification 400×.

## 4 Discussion

The strong positive correlation between Rolando's [17] and Masmali's [18] scales supports the reliability of both classifications and validates their concurrent use, consistent with previous reports in other species [10, 11, 12]. The decision to use both scales was based on Masmali's greater inter‐observer reproducibility, while Rolando's scale remains the most widely used reference for TFT assessment in humans [18]. Additionally, STEPanizer has been validated as a valuable tool to reduce subjectivity in quantitative TFT evaluations [12].

TFT analyses in different domestic species and humans have revealed interspecies variations. In Masmali's scale, healthy humans are most frequently classified as grade 1 (mean 1.5 ± 0.57), whereas individuals with DED are most often classified as grade 2 (mean 2.5 ± 0.52) [25]. In dogs, Type I and grade 1 were most prevalent [10]; in cats, 50% of eyes were classified as Type I [11]; and in horses, 30% as Type I and 51.7% as Type II [12]. The higher prevalence of Types III–IV observed in sheep suggests comparatively lower tear film quality, which may be related to both biological and environmental factors.

Environmental influence is a highly relevant factor for the TFT, especially relative humidity and temperature [24]. Based on the climatic characteristics of the Central‐West region, marked by alternating dry, humid, and high‐temperature periods, tear film instability due to excessive evaporation is expected, potentially impairing ocular surface immune defenses and favoring bacterial proliferation [7, 35, 36]. The combined influence of climate, pen dust exposure, and grazing habits may further contribute to less defined ferning patterns. The temperature (21.5°C) and humidity (49.5%) conditions during sample collection were within the non‐deteriorating range described for ferning patterns [24], minimizing their direct impact on results.

Biological and metabolic differences in the sheep tear film, including lipid, electrolyte, and mucin composition, may also have contributed to interspecies variation. Shamsi et al. [37] identified species‐specific proteins—such as vitelline membrane outer layer protein 1 (VMO‐1) homolog—in sheep and camels but not in humans or cattle, while lipocalin, lysozyme, lactoferrin, immunoglobulins, and serum albumin were common to all species. These differences likely reflect evolutionary adaptations to arid climates [37]. Although age was not statistically correlated with ferning patterns in this study (Figure 4), suboptimal patterns in older animals may suggest a trend toward age‐related tear film instability, warranting investigation in studies with broader age distributions [5, 6, 38, 39].

In sheep, the only previous study specifically addressing tear ferning patterns evaluated the effect of adding various electrolyte solutions to ovine tears in vitro. In that study, baseline TF grades in clinically healthy sheep ranged from approximately 1.7–1.4 on a 0–4 scale and improved to 1.3–0.3 after the addition of divalent electrolytes such as calcium chloride or magnesium chloride, with smaller improvements observed after sodium dihydrogen phosphate or potassium chloride and minimal changes with sodium chloride [16]. In contrast, the present study found a predominance of higher ferning grades (Rolando Types III–IV and Masmali Grades 2–3) in sheep under field conditions, suggesting comparatively lower tear film quality and greater ferning disruption in vivo. These differences may reflect not only environmental factors, including temperature, humidity, and dust exposure, but also species‐ and context‐specific variations in tear electrolyte and protein composition, which can modulate ferning behavior even in clinically healthy animals.

The absence of a significant correlation between TFT and conjunctival histological findings may be explained by the mild intensity of the inflammatory alterations observed. In humans, the GC population can tolerate moderate inflammation without significant functional loss, and tear film stability may be maintained in the presence of low‐to‐moderate inflammatory processes [40, 41]. The predominantly mild lymphocytic infiltrate observed in sheep was likely insufficient to alter conjunctival morphology and tear film composition to a degree detectable by TFT (Figure 6). Furthermore, HE and PAS staining do not detect subtle molecular alterations or changes in mucin secretion quality, and GC counts may have been underestimated as PAS preferentially marks mucous content, leaving already‐discharged cells uncounted [12, 42]. Representative PAS‐positive goblet cells are shown in Figure 7.

**FIGURE 6:**
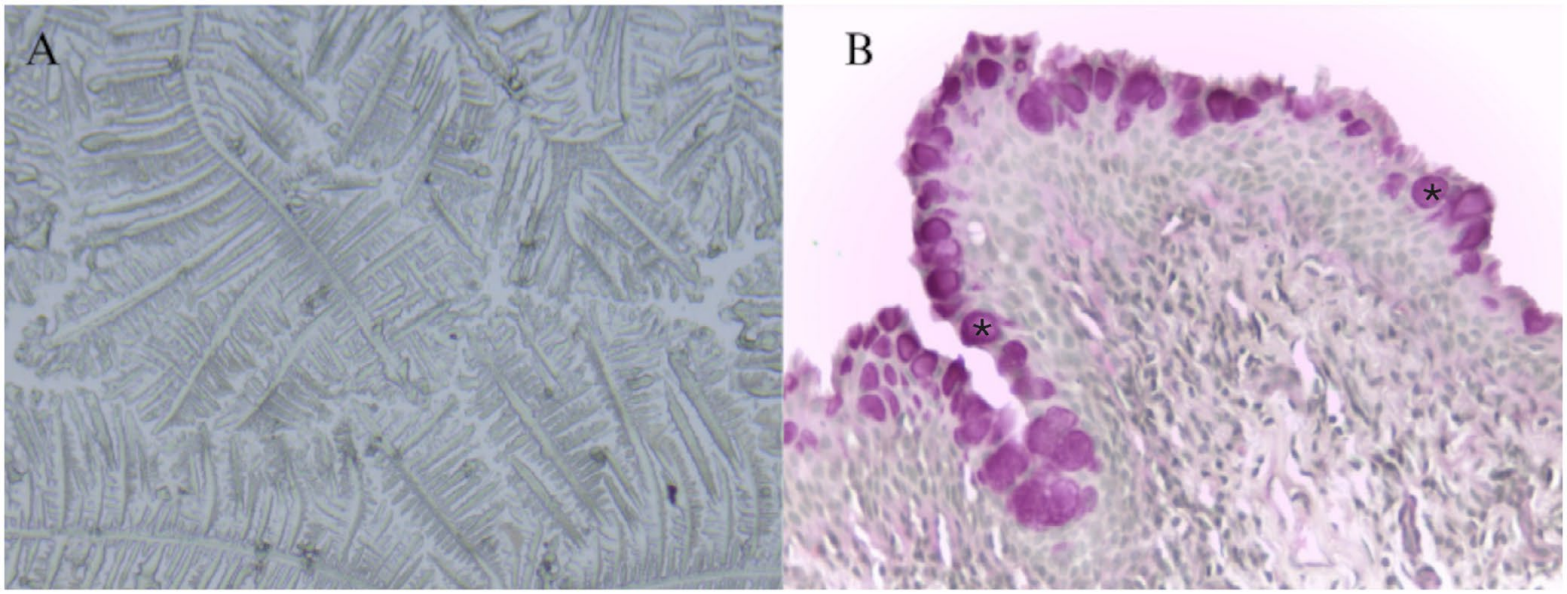
Slides from the same sheep: (A) tear crystal ferning pattern classified as Type II on the Rolando scale and Grade 1 on the Masmali scale (100×); (B) histological section of palpebral conjunctiva stained with PAS, with asterisks (*) indicating goblet cells in the field (400×).

**FIGURE 7:**
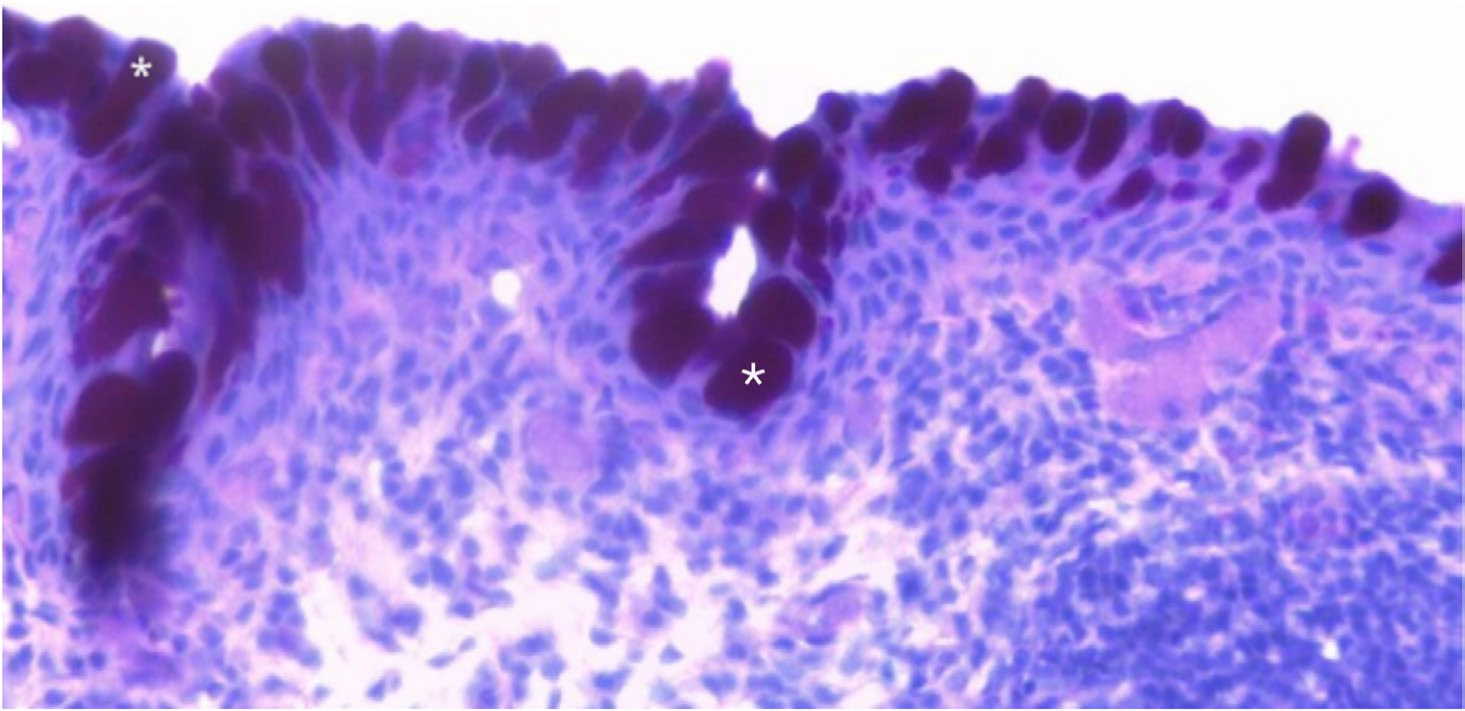
Histological section of palpebral conjunctiva stained with PAS, with asterisk (*) indicating a goblet cell. Magnification 400×.

These results are consistent with findings in horses [12] and healthy dogs [10], where no significant correlations were found between TFT and conjunctival histological parameters. In contrast, human studies have associated tear film deficiency with increased interferon‐γ expression, decreased GC density, and greater conjunctival inflammation [43]. Chronic conjunctival inflammation in humans has been linked to GC reduction [21, 43, 44], predisposing to PTF instability and DED through a dynamic cycle involving cytokine imbalance and GC differentiation [45, 46]. These differences suggest that the relationship between TFT and conjunctival histology may be species‐dependent and influenced by the severity and chronicity of inflammation.

Limitations of this study include its cross‐sectional design, the absence of complementary quantitative tests such as STT and BUT, and data collection restricted to a single season. The temporal restriction reflected strict animal management constraints—sampling had to occur before the breeding season to avoid compromising reproductive performance and to minimize stress in pregnant or lactating ewes. No distinction was made between clinically dry and non‐dry eyes, limiting extrapolation to pathological conditions. Histological analysis also lacked immunohistochemical markers and molecular mucin assessment, which could have detected more subtle tissue alterations.

## 5 Conclusions

This study applied a modern methodological approach combining morphological assessment of tear ferning patterns with stereological quantification, providing reliable and reproducible data for the evaluation of the tear film in sheep. The findings reinforce the potential of the tear ferning test as a practical tool for ocular physiology studies in tropical environments. Future research should expand temporal coverage, include complementary diagnostic methods, and establish species‐specific reference values to enhance the diagnostic applicability of this technique in veterinary ophthalmology.

## Author Contributions

**Aline Stephanie Silva Carvalho:** investigation, writing – original draft, writing – review and editing, methodology, formal analysis, conceptualization, data curation. **Brendha Mendes Ferreira:** investigation, methodology, formal analysis. **Juliano Pereira Terra:** investigation, methodology. **João Vitor Viana Borges:** methodology, investigation. **Fabiano José Ferreira Sant'Ana:** investigation, writing – review and editing, methodology, data curation. **Paulo Henrique Sampaio Silva:** investigation, methodology, formal analysis, data curation. **Paula Diniz Galera:** conceptualization, funding acquisition, methodology, validation, writing – review and editing, project administration, supervision, investigation, data curation.

## Funding

This study was financed in part by the Coordenação de Aperfeiçoamento de Pessoal de Nível Superior—Brasil (CAPES)—Finance Code 001.

## Ethics Statement

This study was reviewed and approved by the Brasília's University Animal Use Ethics Committee (Protocol No. 23106.068874/2024–67). All procedures complied with national animal welfare regulations and followed the Guidelines for Ethical Research in Veterinary Ophthalmology (GERVO). Tear collection was performed using non‐invasive methods, and animal handling was conducted to minimize stress and ensure welfare throughout the study.

## Conflicts of Interest

The authors declare no conflicts of interest.

## Data Availability

The datasets generated and analyzed during the current study are available from the corresponding author upon reasonable request.
